# Ventilator associated pneumonia or ventilator induced pneumonia

**DOI:** 10.1186/s40248-017-0086-3

**Published:** 2017-02-28

**Authors:** Zahid Hussain Khan, Piero Ceriana, Claudio F. Donner

**Affiliations:** 1 Department of Anesthesiology & Intensive Care, Inam Khomeini Medical Center, Tehran University of Medical Sciences, Tehran, 1419733141 Iran; 2 Respiratory Sub-Intensive Care Unit – IRCCS Istituti Clinici Scientifici S.Maugeri, 27100 Pavia, Italy; 3 Mondo Medico di I.F.I.M. srl, Multidisciplinary and Rehabilitation Outpatient Clinic, Borgomanero, NO Italy

**Keywords:** Ventilator, Pneumonia, Associated, Induced

## Abstract

Ventilator associated pneumonia currently in vogue seems to have some pitfalls as far as the nomenclature is concerned and thus it imparts an erroneous impression to the reader. As the driving force is in fact the ventilator, the phraseology should preferably be changed to ventilator induced pneumonia to convey the in depth meaning of the term thus evading the terminology currently in practice. A new and emerging paradigm dealing with all side effects of mechanical ventilation can be helpful to solve this etymological conflict.

Although ventilator associated pneumonia (VAP) has been customarily linked to develop after the use of mechanical ventilation, it appears that the word “association” imparts an erroneous impression as it could wrongly be assumed as an incidental finding, whereas the partnership between a ventilator and pneumonia is in fact an established and unequivocal bondage instead of an incidental or an accidental finding. In its present form, VAP could impart an impression that the pneumonia existed already but by chance accidentally got associated with the ventilator and mechanical ventilation.

Ventilation with high tidal volumes can increase vascular filtration pressures inducing lesions of capillary endothelium, epithelium, and the basement membrane and hence causing lung rupture. Mechanical damage leads to leakage of fluid, protein, and blood into the tissue and air spaces or leakage of air into tissue spaces. These pathologic events are followed by an inflammatory response with the possibility of an impaired defense against an evolving infection. High peak inspiratory volumes and pressures and a high mean airway pressure represent predisposing factors for lung injury. A correct setting of the ventilator during therapeutic ventilation can contribute to prevent overdistension of functional lung units, minimizing lung damage.

Ventilator has been widely accepted as an incriminating factor in the pathogenesis of VAP and regarded as the second most common nosocomial infection in mechanically ventilated patients [1–3], although in recent years the focus has been shifted to the endotracheal tube (ETT) and it has been suggested that VAP could be changed to endotracheal associated pneumonia [4]. The latter name forwarded recently can be discarded on the scientific grounds and evidence that the ETT would fail to cause pneumonia unless that sheer and propelling force of the ventilator is at its back, meaning thereby that it is the ventilator which triggers the process of pneumonia and not the ETT by itself. In reality, it is in fact a microorganism that sets in the seed for the development of pneumonia. If a microorganism did not exist, there would not be any pneumonia. However, mechanical ventilation accelerates and induces pneumonia and causes its progression till it finally reaches a stage that is almost unmanageable. If the driving force of the ventilator had not been there, the microorganism could well have been controlled and so could have pneumonia been overpowered. The ventilator thus inflicts the mortal blow to the lungs in spreading the pneumonia and so should shoulder the brunt of the insult. If that is accepted, then of course this fragile association of ventilator and pneumonia falls to the ground, and is ended of its own accord. Summing up our recollections, the VAP sort of thing does not square with our ideas in this domain. The thought and ingenuity spent when VAP was first introduced can be well appreciated but it looks now as if it has outlived its time and fervor and needs a change, despite the fact this could generate confusion and destabilization in a part of the scientific community deeply accustomed to this terminology and phraseology.

The term association can be very fragile and can be easily shattered and reduced to shambles which we commonly encounter in our daily life and dealings. Association between people and organizations, no matter how strong it may be, gets dismantled very easily, reinforcing our notion expressed above that ‘association’ in fact is a weak bond as far as its application with the ventilator and pneumonia is jointly concerned. As the term implies, an association can be loose and subject to breakage and thus cannot be correctly applied to VAP and bracketed with pneumonia which is a concrete pathological finding and reinforced by X-ray findings and evidences.

It seems that ‘ventilator’ associated or bracketed with pneumonia is just lumping together two terms wrongly or mistakenly blended together without that meaning that it was intended and destined to convey.

Having said that, it is felt that the word ‘associated’ could be replaced with the word “induced” and the nomenclature changed from VAP to ventilator-induced pneumonia (VIP) and pronounced as VIP breaking into syllables, because it is the ventilator which is the impelling and generating force in the causation of pneumonia. Since induction means that the occurrence of pneumonia is in fact promoted or stimulated by the ventilator, the loose term of ‘association’ that obviously does not provide a good outfit, can be safely discarded and replaced by the term ‘induced’.

Since pneumonia does not exist at the start and is induced by the ventilator, the word “induced” would be a better apparel and would appear more appealing and acceptable as far as the intensivists are concerned and would prompt them to use the ventilator more cautiously, as it in fact works as a generating force in the causation of pneumonia. A further reinforcement to this concept comes from the microbiological evidence that pneumonia during mechanical ventilation is closely connected to the colonization of the oral cavity and with the subsequent aspiration of these pathogens that leak around the cuff of the tracheal tube and migrate up to the distal airways [5]. A study comparing the microbiological agents cultured from both the oral cavity and the bronchoalveolar lavage (BAL) of patients with pneumonia during mechanical ventilation confirmed that the bacteria isolated from the mouth were the same that had been isolated from the lung [6]. Besides, the biofilm on the tracheal tube must be considered [7] as a means to facilitate the diffusion of bacteria along the airways.

## Recent insight into the concept of VAP or VIP

Incidence of pulmonary infections in patients intubated and undergoing mechanical ventilation ranges between 9 and 27% [1]. This wide range has different explanations: there has been a growing awareness in the last years about the difficulty in diagnosing pneumonia in mechanically ventilated patients due to the high inter-observer variability and the subjective evaluation of some diagnostic criteria like chest X-ray imaging, sputum evaluation, and in some cases even auscultation [8]. Second, there is more attention now than in the past in defining the real extent of the airway infection, since when the infectious process is confined up to the distal airways but without a true parenchimal involvement, the term ventilator-induced tracheobronchitis is preferred [9]. Third, the clinical presentation and context in which VAP or VIP must be diagnosed can be very different like after open-heart surgery, chest trauma or lung transplantation. Therefore, setting uniform and homogeneous criteria for the definition of pneumonia during invasive mechanical ventilation can be very challenging.

A possible solution to the “etymological dilemma” between VAP or VIP can be found in a recent change of paradigm about pulmonary infectious events during mechanical ventilation: it has been proposed by Klompas and Kalil [10, 11] to overcome the old concept of VAP in favor of the new concept of ventilator-associated events (VAE). This embraces all the complications related to mechanical ventilation broadening the horizon of possible consequences beyond those infection-related. A VAE is defined as at least two days of stable or decreasing ventilator setting followed by at least two days of increased ventilator setting. The change in ventilator setting implies in particular an increase in positive end expiratory pressure (PEEP) of at least 3 cmH_2_0 per day for two consecutive days and of inspired fraction of oxygen of 20% for two days relative to the preceding days. The advantage of this new definition is that the clinician’s attention is shifted from the simple problem of pneumonia to all unwanted respiratory events connected to mechanical ventilation (atelectasis, ARDS, fluid overload). Respiratory infections become part of this definition under the name of infection-related ventilator-related complications (IVACs).

## Abbreviations

BAL: Bronchoalveolar lavage
ETT: Endotracheal tube
IVACs: Infection-related ventilator-related complications
PEEP: Expiratory pressure
VAE: Ventilator-associated events
VAP: Ventilator associated pneumonia
VIP: Ventilator-induced pneumonia

## References

[1] American thoracic Society, Infectious Diseases Society of America (2005). Guidelines for the management of adults with hospital-acquired, ventilator-associated, and healthcare-associated pneumonia. Am J Respir Crit Care Med.

[2] Chastre J, Fagon JY (2002). State of the art: ventilator-associated pneumonia. Am J Respir Crit Care Med.

[3] Hunter JD (2012). Ventilator associated pneumonia. BMJ.

[4] Pneumatikos JA, Dragoumanis CK, Bouros DE (2009). Ventilator-associated pneumonia or endotracheal tube-associated pneumonia? An approach to the pathogenesis and preventive strategies emphasizing the importance of endotracheal tube. Anesthesiology.

[5] Cook D, De Jonghe B, Brochard L, Brun-Buisson C (1998). Influence of airway management on ventilator-associated pneumonia: evidence from randomized trials. JAMA.

[6] Bahrani-Mougeot FK, Paster BJ, Coleman S, Barbuto S, Brennan MT, Noll J, et al. Molecular analysis of oral and respiratory bacterial species associated with ventilator-associated pneumonia. J Clin Microbiol. 2007;45(5):1588–93. 10.1128/JCM.01963-06 PMC1865865 17301280

[7] Inglis TJ, Millar MR, Jones JG, Robinson DA (1989). Tracheal tube biofilm as a source of bacterial colonization of the lung. J Clin Microbiol.

[8] Stevens JP, Kachniarz B, Wright SB, Gillis J, Talmor D, Clardy P, et al. When policy gets it right: variability in US hospitals’ diagnosis of ventilator associated pneumonia. Crit Care Med. 2014;42:497–593. 10.1097/CCM.0b013e3182a66903 24145845

[9] Palmer LB (2015). Ventilator-associated tracheobronchitis and pneumonia. Lancet Respir Med.

[10] Klompas M (2013). Complications of mechanical ventilation-the CDCs new surveillance paradigm. N Engl J Med.

[11] Klompas M, Kalil AC (2014). The “last breath” of the ventilator-associated pneumonia surveillance definition. Crit Care Med.

